# Autologous Peripheral Blood Stem Cell Therapy for Chronic Achilles Tendinopathy: Report of Three Cases

**DOI:** 10.5704/MOJ.2207.022

**Published:** 2022-07

**Authors:** KY Saw, SF Low, A Ramlan, A Dawam, YC Saw, CSY Jee

**Affiliations:** 1 Department of Orthopaedic Surgery, Kuala Lumpur Sports Medicine Centre, Kuala Lumpur, Malaysia; 2 Department of Radiology, Kuala Lumpur Sports Medicine Centre, Kuala Lumpur, Malaysia; 3 Stem Cells Research and Development, Kuala Lumpur Sports Medicine Centre, Kuala Lumpur, Malaysia

**Keywords:** chronic Achilles tendinopathy, tendinitis, peripheral blood stem cells, VISA-A scores

## Abstract

The treatment of chronic Achilles tendinopathy (CAT) remains challenging. We report three cases of CAT treated with autologous peripheral blood stem cells (PBSCs), following principles developed for chondrogenesis of the knee joint. Outcome measurement with a minimum of one and a half years follow-up showed significant improvement of Victorian Institute of Sport Assessment-Achilles questionnaire (VISA-A) scores, with reduction of tendon thickness and inflammation on MRI scan.

## Introduction

Chronic Achilles tendinopathy (CAT) is debilitating and causes prolonged pain and disability. As the Achilles tendon is relatively avascular with a slow healing potential, overuse injury leads to repeated micro-tears of the tendon, resulting in chronic tendinopathy. The ideal treatment of CAT remains elusive. Non-surgical and surgical methods have not been entirely curative and often relapse^1^.

We pioneered chondrogenesis with autologous peripheral blood stem cells (PBSCs), addressing massive knee chondral defects^2^. Following the principles developed for chondrogenesis, we report the results of applying PBSCs therapy to address CAT.

## Case Reports

**Fig. 1:**
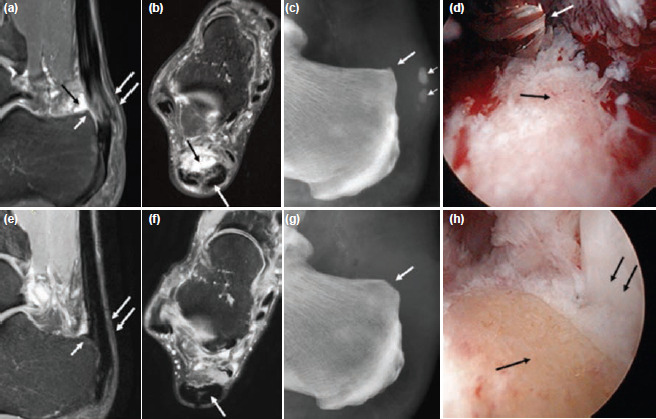
(a) Sagittal and (b) axial proton density fat suppressed (PDFS) MR images of the right ankle showing Haglund's triad with Haglund deformity (short white arrow), retrocalcaneal bursitis (black arrow) and insertional Achilles tendinopathy (double white arrows). Hyperintense signal and interstitial split tears were noted at the distal Achilles tendon (long white arrows). (c) Lateral ankle radiograph shows Haglund deformity (white arrow) and calcifications in the retro-Achilles bursa (small arrows). (d) Posterior ankle arthroscopic view from the medial portal showing the Haglund deformity (black arrow) and a 4-mm arthroscopic burr (white arrow) from the lateral portal. (e, f) Similar MR images as in (a, b) six years following surgery with burring of previous Haglund deformity (short white arrow), showing repair and regeneration of the Achilles tendon with reversal of the pathology to signal normality (long white arrows). (g) Lateral ankle radiograph following arthroscopic burring of the Haglund deformity (white arrow) and removal of the calcifications in the retro-Achilles bursa. (h) Arthroscopic view similar to (d) following arthroscopic burring of the Haglund deformity (black arrow) revealing the previously impinged Achilles tendon (double black arrows).

The first case was a 59-year-old man with a five years’ history of CAT, following unsuccessful conservative treatment. MRI scan and radiographs showed Haglund's triad with Haglund deformity, retrocalcaneal bursitis and insertional Achilles tendinopathy (Fig. 1a and 1b). This was associated with calcifications in the retro-Achilles bursa (Fig. 1c). Autologous PBSCs were harvested before surgery. The details of the harvesting procedure and cell preparation are outlined in our previous publication^2^. He underwent surgery with removal of the calcified loose bodies in the retro-Achilles bursa, arthroscopic burring of the Haglund deformity (Fig. 1d) and multiple needling (23G needle) into the inflamed Achilles tendon. Immediately after the multiple needling, 8mL aliquot of thawed cryopreserved PBSCs were mixed with 2mL hyaluronic acid (HA) [Hyalgan; Fidia Farmaceutici, Abano Terme, Italy] and injected into the inflamed Achilles tendon. At four subsequent weekly intervals, the identical PBSCs plus HA mixture were injected into the same area under aseptic conditions while sedated. Physiotherapy with ultrasound, transcutaneous electrical nerve stimulation and joint mobilisation commenced one day after surgery and he mobilised with a lower leg walker (Aircast ankle boot), progressing from partial to full weight bearing in six weeks. Muscle strengthening and stationary bike cycling commenced after six weeks. At month three and six following surgery, three additional weekly injections comprising 4mL thawed cryopreserved PBSCs and 2mL HA were given. MRI scan following surgery showed repair and regeneration of the CAT with reversal of the pathology to signal normality (Fig. 1e-1h).

**Fig. 2:**
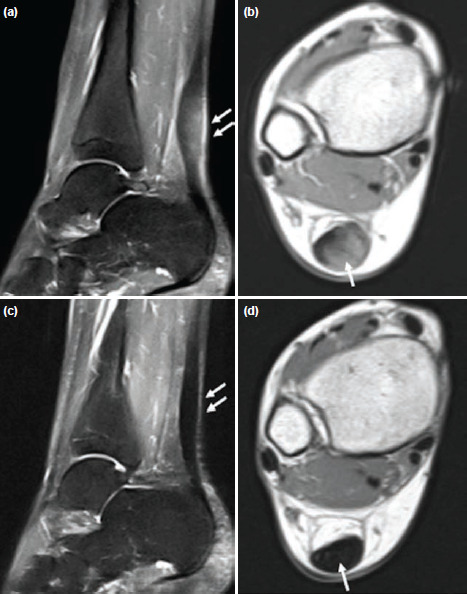
(a) Sagittal short tau inversion recovery (STIR) MR image and (b) axial T2W MR image of the right ankle showing non-insertional Achilles tendinosis with fusiform thickening and hyperintense signal of the Achilles tendon (white arrows). (c) Sagittal STIR MR image and (d) axial T2W MR image showing the Achilles tendon resuming its normal signal intensity and appearing less thickened compared to previous study (white arrows) at six months following multiple needling and a five-weekly injections of PBSCs plus HA.

The second case was a 41-year-old woman with one year’s history of pain and swelling over the right Achilles tendon. MRI scan as shown (Fig. 2a and 2b) revealed non-insertional Achilles tendinopathy with fusiform thickening associated with hyperintense signal. The fusiform swelling with inflammatory changes over the Achilles tendon resolved following multiple needling into the Achilles tendon and a five-weekly injection of PBSCs plus HA (Fig. 2c and 2d). The pre-operative preparation, intra-operative injections and post-operative physiotherapy regime were similar to the first case. No further additional injections at months three and six were required as compared to the first case because of significant clinical and radiological improvement.

The third case was a 49-year-old man with a three years’ history of CAT. Sagittal and axial proton density fat suppressed MR image of the right ankle showed non-insertional Achilles tendinopathy with intrasubstance hyperintense signal. This was treated with procedures and post-operative regime similar to the second case. Cases 2 and 3 were non-insertional CAT, hence no burring of the Haglund deformity was required.

Victorian Institute of Sport Assessment-Achilles questionnaire (VISA-A) score was used as patient outcome scores for each of the cases reported here at final follow-up (Table I). The VISA-A score has been shown to be reliable for assessing functional outcome relating to CAT treatment^3^. It is based on a numeric score of 0 to 100 points, with asymptomatic subjects expected to score 100 points. Our three cases showed post-operative scores above 70, indicating scores comparable to healthy subjects^4^. There were no documented infections or major adverse events.

**Table I:** Patient demographics and VISA-A scores from pre- to post-surgical follow-ups in all three cases.

Case	Gender	Age at surgery (years)	Duration of symptoms (months)	Period of follow-up (years)	Pre-surgical	VISA-A scores Post-surgical	Improvement
1	Male	59	60	6.0	31	100	69
2	Female	41	12	9.5	41	97	56
3	Male	49	36	1.5	38	72	34
Mean	-	50	36	5.7	37	90	53

## Discussion

Autologous PBSCs therapy is a viable option for the treatment of CAT. Objective evaluation by MRI scans assessed the repair area non-invasively and documented satisfactory healing process, with no evidence of adverse abnormalities. This was accompanied by significant improvement of the VISA-A scores in all three cases.

**Fig 3:**
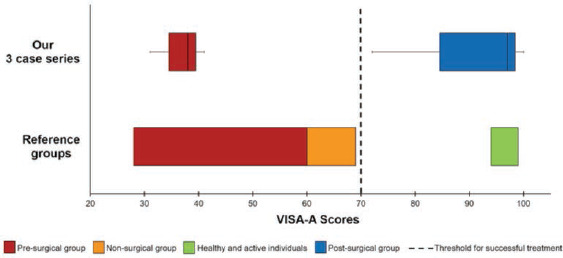
Diagram showing VISA-A scores of different groups; namely pre-surgical, non-surgical, healthy and active individuals, and post-surgical. The upper bars represent the scores from our three-case series. The lower bars indicate reported scores, whereby the dotted line is the threshold score that indicate treatment success (Robinson *et al*, 2001)^4^.

The mean VISA-A score in non-surgical patients with Achilles tendinopathy was 64 (59-69) with a mean age of 42.3 years, in pre-surgical patients 44 (28-60) with a mean age of 44.3 years, and in control subjects it exceeded 96 (9499)^4^. Following treatment, a score above 70 can be considered satisfactory (Fig. 3)^4^. An overall increase of VISA-A score of 28.9 points showed significant improvement as reported by Madhi *et al* in systematic reviews^1^.

Comparing with the VISA-A score in pre-surgical patients, the mean age of our three patients was slightly older at 49.6 as compared to 44.3 years, the mean duration of symptoms was longer at 36 as compared to 19.2 months and the mean pre-surgical score was lower at 36.6 as compared to 44 points^4^. All our three cases showed scores above 70 with an overall increase beyond 28.9 points following surgery, indicating scores comparable to healthy subjects^4^. Two patients (Cases 1 and 2) achieved a score of 100 and 97 points at a follow-up period exceeding six and nine years, respectively (Fig. 3 and Table I). This suggests that PBSCs therapy has the ability to be curative and regenerative in nature and that long term results are sustainable.

The use of platelet-rich plasma for treating CAT has gained tremendous interest lately, but high level of evidence studies did not show a significant efficacy and the evidence supporting it is limited^1^. Anz *et al*^5^ recently concluded that PBSCs showed proliferative potential and were pluripotent. Thus, PBSCs has the ability to repair and regenerate all the native cellular types of the musculoskeletal system (MSK).

Applying the same principles we developed for knee chondrogenesis with PBSCs^2^, we believe that the pathway to the repair and regeneration of CAT requires three key principles, namely: (1) Creating fresh injury (by multiple needling), (2) Cellular therapy with multiple PBSCs plus HA injections (to provide enough cells to create matrix substance, release growth factors and differentiate into various components of the MSK), and (3) Functional stimulation with tailored physiotherapy (allows the stem cells to differentiate in the native functional environment, thus regenerating the desired tissue type).

As seen in our three reported cases, autologous PBSCs seem to be a promising orthobiologics for the repair and regeneration of CAT and likely applicable to the other aspects of the MSK^5^. A future randomised controlled clinical trial along the direction we have embarked similar to knee chondrogenesis with PBSCs^2^ would be ideal to further validate this early concept.
